# Hydrogen peroxide modifies both activity and isoforms of acetylcholinesterase in human neuroblastoma SH-SY5Y cells

**DOI:** 10.1016/j.redox.2017.04.004

**Published:** 2017-04-04

**Authors:** Alba Garcimartín, M. Elvira López-Oliva, M. Pilar González, Francisco J. Sánchez-Muniz, Juana Benedí

**Affiliations:** a Departamento de Farmacología, Facultad de Farmacia, Universidad Complutense de Madrid, Madrid, Spain; b Sección Departamental de Fisiología, Facultad de Farmacia, Universidad Complutense de Madrid, Madrid, Spain; c Departamento de Nutrición y Bromatología I, Facultad de Farmacia, Universidad Complutense de Madrid, Madrid, Spain

**Keywords:** Acetylcholinesterase, Hydrogen peroxide, Alternative splicing, Cell culture, Cell death

## Abstract

The involvement of cholinergic system and the reactive oxygen species (ROS) in the pathogenesis of some degenerative diseases has been widely reported; however, the specific impact of hydrogen peroxide (H_2_O_2_) on the acetylcholinesterase (AChE) activity as well as AChE isoform levels has not been clearly established. Hence, the purpose of present study is to clarify whether H_2_O_2_ alters these parameters.

Human neuroblastoma SH-SY5Y cells were treated with H_2_O_2_ (1–1000 µM) for 24 h and AChE activity and AChE and cytochrome c levels were evaluated. AChE activity was strongly increased from 1 µM to 1000 µM of H_2_O_2._ The results of the kinetic study showed that H_2_O_2_ affected Vmax but not Km; and also that H_2_O_2_ changed the sigmoid kinetic observed in control samples to hyperbolic kinetic. Thus, results suggest that H_2_O_2_ acts as an allosteric activators. In addition, H_2_O_2_, (100–1000 µM) reduced the total AChE content and modified its isoform profile (mainly 50-, 70-, and 132-kDa)·H_2_O_2_ from 100 µM to 1000 µM induced cytochrome c release confirming cell death by apoptosis. All these results together suggest: a) the involvement of oxidative stress in the imbalance of AChE; and b) treatment with antioxidant agents may be a suitable strategy to protect cholinergic system alterations promoted by oxidative stress.

## Highlights

• H_2_O_2_ impact on AChE structure from SH-SY5Y cells, acting as an allosteric effector.
• H_2_O_2_ decreased AChE levels and changed AChE isoform profile from SH-SY5Y cells.
• Oxidative stress could promote disturbances in cholinergic system.

## Graphical abstract

**Figure:**
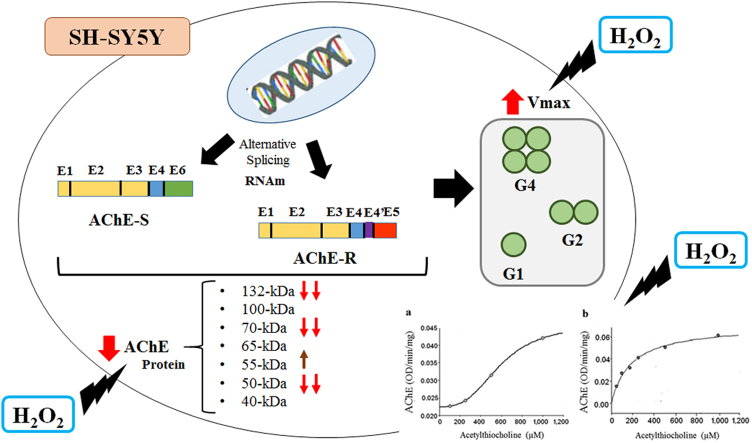

## 1 Introduction

Acetylcholinesterase (AChE) (EC 3.1.1.7) is the enzyme responsible for the termination of cholinergic neurotransmission [1]. Several neurological disorders, chiefly Alzheimer disease (AD), are associated with abnormal expression and activity of this enzyme [2], [3]. In fact, AChE inhibitors (AChEIs) are used as gold standard treatment in Alzheimer disease (AD) [4]. Although the true benefit of these drugs is currently coming into question [5].

Alternative splicing gives rise to three isoforms of AChE from a single gene. Such isoforms differ in their C-termini [6], leading to different post-translational modifications [7]. Nevertheless, all of them retain the capacity to hydrolyze Ach [8]. These isoforms are known as AChE-R, AChE-E and AChE-S [9]. AChE-E is found in erythrocytes, while AChE-R and AChE-S are the splice variants present in the brain. AChE-S appears as monomers (G1) and also as dimers (G2) or tetramers (G4) formed by assembly with a proline-rich membrane anchor [10]. The AChE-R is related to external stressors [9] and its soluble monomeric subunit seems to be able to associate to form complexes without disulphide bounds [11].

Although reactive oxygen species (ROS) are continuously produced in the cells [12], it is widely accepted that oxidative stress plays an important role in many chronic disease, including neurodegenerative diseases. In fact, aging, one of the main risk factors of these diseases, is associated with ROS accumulation [13]. Moreover, ROS increase is detected in the early stages of such diseases, even when others characteristic features remain unchanged, suggesting that oxidative stress is implicated in their etiology [14]. For example, oxidative stress has been found in AD models even when Aβ levels remained unchanged [15]. Regarding AD, both, oxidative stress and AChE have been linked to Aβ generation.

Effect of H_2_O_2_ on AChE has been studied in different models. Thus, Schallreuter et al. [16] found that H_2_O_2_ a high concentrations, which is characteristic of epidermal vitiligo disease, regulates AChE activity in a dose-dependent manner, via oxidation of methionine, cysteine and tryptophan all residues closely related to the active centre. Also, Zhang et al. [17] observed a H_2_O_2_-dependent increase of AChE expression via transcriptional JNK; and Molochkina et al. [18] showed that H_2_O_2_ modifies AChE activity by modification of the cellular membrane. Despite all of these works, no link between oxidative stress and AChE has been identified in human brain cells. In this context, it would be interesting to know the effect of ROS on cholinergic system markers from undifferentiated SH-SY5Y neuroblastoma cells; because, although these cells are not specifically cholinergic, they present AChE enzyme as has been previously demonstrated by Filograna et al. [19]. Although there are some studies about the action of pro-oxidant substances on AChE of different cell lines, to the best of our knowledge the direct effect of oxidative stress on the levels of AChE and AChE activity in human brain cells remains unclear. In the present study it was hypothesized that H_2_O_2_ produces a specific effect on AChE in the human neuroblastoma SH-SY5Y cell line, modifying the levels and isoform profile of this enzyme.

H_2_O_2_ is particularly interesting because: a) it is the most stable ROS [20]; b) it passes through cell membranes [21]; and c) it does not act just as an extracellular messenger but also as an intercellular one, causing alteration of several proteins [22].

The present study in SH-SY5Y human neuroblastoma cell line aims to determine the effect of H_2_O_2_ on AChE activity and isoform profiles.

## 2 Materials and methods

### 2.1 Reagents/Materials

Dulbecco's modified Eagle's medium (DMEM), fetal bovine serum (FBS), 0.25% trypsin-EDTA, and penicillin/streptomycin mixture were purchased from GIBCO–BRL (Grand Island, NA, USA)·H_2_O_2_, acetylthiocholine iodide, 5,5-dithio-bis(2-nitrobenzoic) acid (DTNB), aprotinin, leupeptin and phenyl-methane-sulfonyl fluoride (PMSF) and Triton X-100 were obtained from Sigma Chemical Co.(St. Louis, MO, USA). Anti-acetylcholinesterase antibody (N-19) (sc-6431), anti-cytochrome c (sc-8385), and anti β-actin antibody (sc-47778) were supplied by Santa Cruz Biotechnology, Quimigen (Madrid, Spain). PVDF membrane and ECL select were purchased from GE Healthcare (Madrid, Spain). Other chemicals were reactive grade products from Merck (Darmstadt, Germany).

### 2.2 Cell culture

Human SH-SY5Y (ATCC CRL-2266) neuroblastoma cells were cultured in DMEM with 10% (v/v) FBS and 100 U/mL penicillin/streptomycin at 37 °C in a 5% CO_2_ and 95% air humidified atmosphere. When cells reached 80–90% confluence were sub-cultured on 100 mm^2^ culture dishes with 10% (v/v) FBS. 24 h later cells were changed to media with 1% FBS and treated with or without H_2_O_2_ (1–1000 μM) during 24 h·H_2_O_2_ was freshly prepared from 30% stock solution prior to each experiment. The control cells were kept in media without H_2_O_2_.The experiments were carried out between 3 and 15 passages.

### 2.3 Estimation of protein

The protein concentration was determined in cell extracts according to Bradford method [23], using bovine serum albumin as standard. The cells were lysed with the buffer used to measure the AChE activity, pelleted and centrifuged (Centrifuge 5804R, Eppendorf, Hamburg, Germany) at 13,0000*g* during 15 min. The protein content was determined on supernatants.

### 2.4 Determination of activity and kinetic parameters of AChE

AChE activity was determined by Ellman's colorimetric method [24] based on the following reactions:

$$\begin{array}{c} \mathrm{Acetylthiocholine}\;\;\overset{\mathrm{AChE}}{⟶}\;\mathrm{Thiocholine}+\mathrm{Acetate}\\ \mathrm{Thiocholine}+\mathrm{DTNB}\to 5-\text{thio}-2-\text{nitrogenzoate}\;\mathrm{anion} \end{array}$$

In these experiments we have used SH-SY5Y extracts as AChE source. Cells were seeded in 100 mm^2^ dishes (2×10^6^ cells) and treated during 24 h with the indicated H_2_O_2_ concentrations. The cells extracts (enzyme source) were prepared homogenizing the cells with 0.1 M KH_2_PO_4_/K_2_HPO_4_ (pH 8.0), containing 0.1% (v/v) Triton X-100, and centrifuging at 13,000*g* for 5 min. Supernatants (50 µl) were mixed with 4 mM of DTNB, increasing concentrations of Acetylthiocholine (50–1000 µM), and phosphate buffer 0.1 M, pH=8. The final volume was 205 µl. The yellow product of this reaction was measured spectroscopically at 412 nm. (LT-4000, Microplate Reader, Labtech International Ltd, United Kingdom), every minute during 10 min. Michaelis-Menten constant (*K*_*m*_) and maximum velocity (*V*_*max*_) were determined by Lineweaver– Burk plot method [25], using the inverse of activity (DO/min/mg protein) and the inverse of substrate concentration (µM). The activity was expressed in OD/m/mg protein.

### 2.5 Effect of H_2_O_2_ on AChE activity from control cell extracts

In this experiment cell culture was performed slightly different. The cells were seeded in 100 mm^2^ dishes (2×10^6^ cells) with 10% FBS media for 24 h. After that time the plates were not treated with H_2_O_2_. Instead of that, they were kept with 1% FBS media during 24 h, as we did with the control cells of the rest of experiments. The cells extracts were obtained as described above (Section 1.4), homogenizing the cells with the buffer and centrifuging at 13,000*g* for 5 min. The treatment was carried out once the supernatants were isolated·H_2_O_2_ (1, 400 and 1000 µM final concentration) was mixed with the cell extract and incubated for 0, 15 or 30 min. In this experiment the control was the extract of the cells without adding H_2_O_2_.

Afterwards, the enzymatic AChE activity was measured as indicate above (section 1.4). Finally, AChE activity defined as punctual velocity (Vp) was normalized to the amount of protein of the extracts and expressed as OD/min/mg protein.

### 2.6 AChE and cytochrome c levels

The protein content was assayed by Western Blot following Garcimartín et al. protocol [26]. SH-SY5Y cells were plated on 100 mm dishes and treated with H_2_O_2_ (1–000 μM) for 24 h. To prepare the samples for AChE analyses the cells were lysed with buffer Tris-HCl (pH 7.4), 1% Triton X-100, 10 mM EDTA, 50 mM NaCl and enriched with aprotinin (2 μg/mL), leupeptin (5 μg/mL) and PMSF (1 mM) as protease inhibitors. After that they were centrifuged at 13,000*g* for 5 min at 4 °C to remove the nuclei and cellular debris (Centrifuge 5804R, Eppendorf, Hamburg, Germany). The samples were manipulated in the same way that the SH-SY5Y cells. On the other hand, to prepare the cytochrome c sample subcellular fractionation was needed [26]. The cells were harvested in hypotonic buffer (10 mM Tris-HCl (pH 7.4), 1 mM sodium vanadate and protease inhibitors) and incubated on ice. The lysates were centrifuged at 800*g* for 5 min at 4 °C to remove the nuclei and cellular debris. The resulting supernatants were further centrifuged at 15,000*g* for 15 min at 4 °C to obtain the supernatant fraction (cytosol). Before starting, 5x-Loading buffer (0.5 M Tris-HCl (pH 6.8), 20% v/v glycerol, 10% w/v SDS, 5 mM β-mercaptoethanol and 0.01% bromophenol blue) was added to each sample and incubated for 5 min at 95 °C. Equal amounts of protein (30 μg) were separated at 150 V in 10% (v/v) polyacrylamide gel (SDS–PAGE) and, after migration, transferred to polyvinylidene fluoride (PVDF) membrane (GE Healthcare, Madrid, Spain) at 400 mA for 1 h, at 4 °C. All membranes were blocked by 5% (w/v) non-fat dry milk for 1 h at room temperature. For immunodetection, membranes were incubated overnight at 4 °C with the primary antibody, anti-AChE (1/1000) or anti-cytochrome c (1/1000), followed by incubation with peroxide-conjugated secondary antibodies for 1 h at room temperature (Santa Cruz Biotechnology, Quimigen, Madrid, Spain). Blots were developed by enhanced chemiluminescence (ECL select; GE Healthcare, Madrid, Spain) according to the manufacturer's instructions. Anti β-actin antibody (1/2000) was used as loading control.

### 2.7 Quantification of protein levels and AChE isoforms

The quantification of the protein levels was made using Image Quant 5.0 software. Each band was measured individually, removing the background signals, and expressed as densitometry unit respect to beta-actin (densitometry/beta actin). To know the content of the whole AChE protein, the corrected data of all bands were added up and considered as the 100% of total AChE protein. The specific contribution of each band to global AChE content was expressed as the percentage of each band (band densitometry × 100/ total densitometry AChE protein).

### 2.8 Principal component analysis

PCA test was applied to understand possible associations between the different bands detected in western blot, which represent the isoforms of AChE, and the kinetic parameters. Ten different variables were tested, seven, related to percentage of isoforms (132-, 100-, 70-, 65-, 55-, 50-, 40-kDa bands), two about kinetic parameters (Vmax and km). PCA create new variables (principal components (PCs)) containing the initial variables which correlate among them. Data number included in PCA was higher than five times (>5x) the number of variables.

### 2.9 Statistical analysis

Data were expressed as mean ±SEM from two independent experiments using different cultures. Each experiment was performed at least in triplicate with different cell batches (total 6–12 measurement/condition). Statistical analyses were performed using SigmaPlot 11.0 software. Data were tested by One Way ANOVA followed by the Bonferroni test. Differences were considered significant at *P*<0.05. SPSS version 19.0 statistical analysis package (SPSS, Inc., Chicago, IL, USA) was used for the PCA test and linear correlations.

## 3 Results

### 3.1 Effect of H_2_O_2_ on AChE activity

**Fig. 1:**
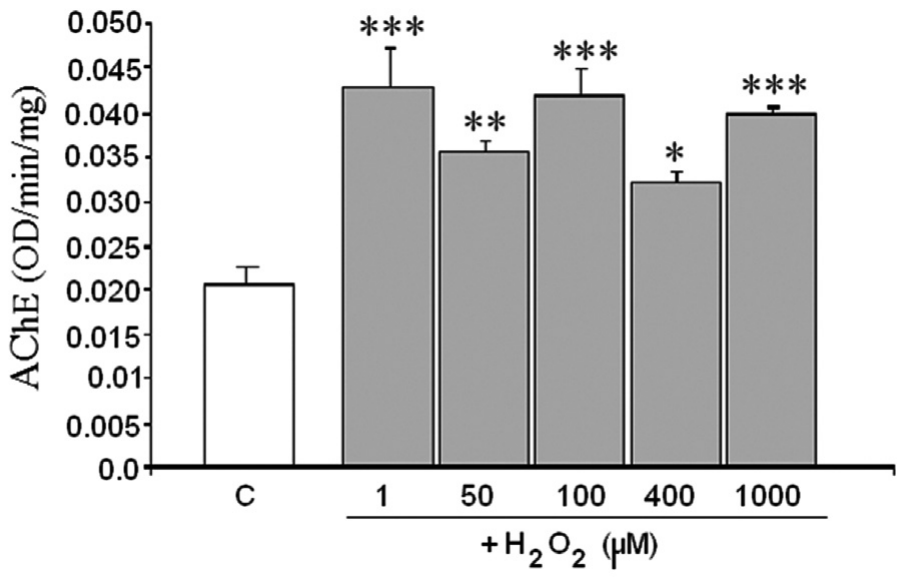
Effect of different concentrations of H_2_O_2_ on AChE activity from SH-SY5Y as enzyme source. Cells were treated during 24 h with 1, 50, 100, 400 and 1000 µM of H_2_O_2_. Control condition (C) is referred to untreated cells. AChE activity was determined by the Ellman's method, using acetylthiocholine (250 μM), and expressed in OD/m/mg protein. Results are means ±SEM of cells from three different cultures each one performed in triplicate. (*) = statistical signification with respect to control. (*) = p<0.05, (**) = p<0.01 and (***) = p<0.001.

As some neurodegenerative diseases have been related with changes in AChE activity along with an increase in oxidative stress, we measured the AChE activity from SH-SY5Y cells which were used as enzyme source. The activity was test in control cells (untreated cells) and in cells after 24 h treatment with H_2_O_2_. The H_2_O_2_ (1–1000 μM) promoted an increase in AChE activity. The highest effect was produced at 1 μM and it was kept until 1000 μM (Fig. 1).

Such an effect on AChE activity could be caused by direct action of the H_2_O_2_ on the structure of the enzyme or by an alteration of the AChE levels, and therefore three different experiments were carried out to ascertain the mechanism involved: a) AChE kinetic parameters evaluated in control cells and after treating the cells during 24 h with different concentrations of H_2_O_2_; b) AChE activity after the addition of H_2_O_2_ to control cell extracts; and c) measure of AChE levels by western blot analysis in control cells and in cells subsequent to the 24 h treatment with H_2_O_2_.

### 3.2 Action of H_2_O_2_ on kinetic parameters of AChE

**Fig. 2:**
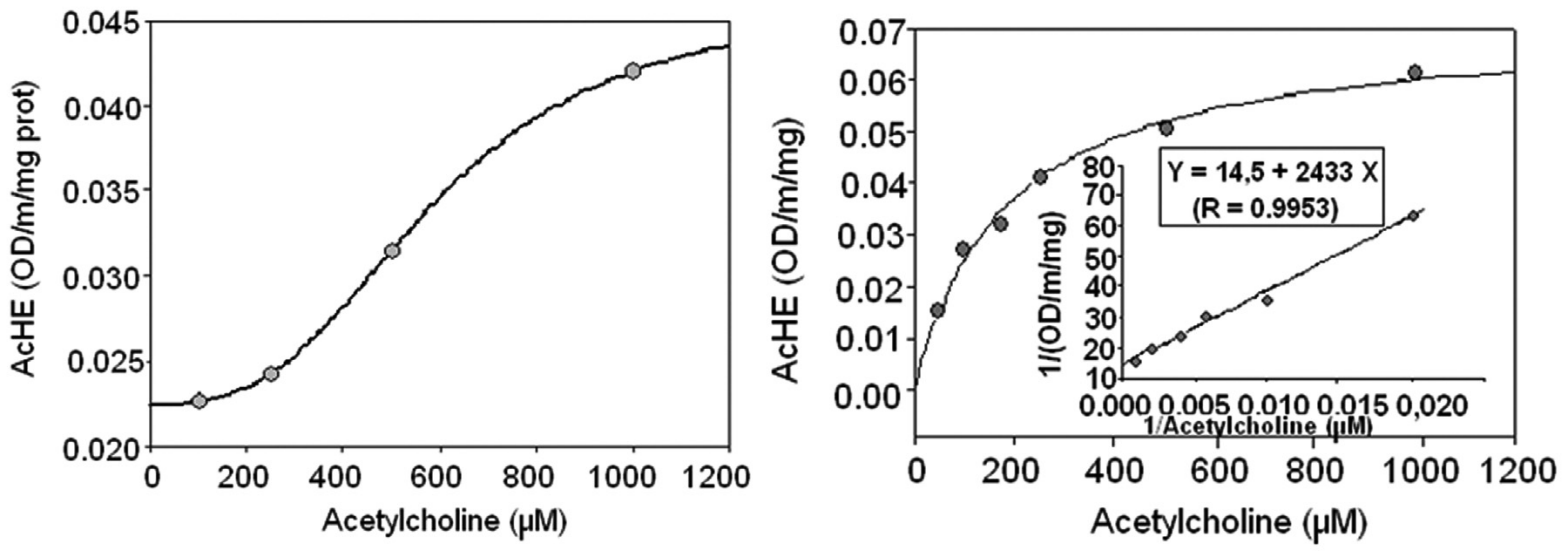
Representation of acetylcholinesterase (AChE) activity vs. acetylthiocholine concentration. Kinetic analysis was performed by the Ellman's method using different concentration of substrate and SH-SY5Y extracts as enzyme source. Enzyme behavior was obtained using the SigmaPlot analysis which evaluated whether the data are adjustable to sigmoidal or hyperbolic adjusts. **(a)** Sigmoidal adjustments typical of control cells and those treated with 1 μM of H_2_O_2_; and **(b)** hyperbolic adjustment characteristic of cells treated with H_2_O_2_ (50–1000 μM). Results are means ±SEM of cell from two different cultures, each one performed in triplicate.

The plot of AChE activity vs acetylthiocholine (substrate) concentration allowed us to further characterize the enzyme and helped to know the enzyme behavior (Fig. 2).

The AChE activity of control cells (kept 24 h in 1%FBS media without any treatment) and those treated with 1 µM of H_2_O_2_ showed an evident sigmoid adjustment with a Hill coefficients of 3.0±0.4 (control cells) and 2.5±1.5 (1 µM H_2_O_2_) (Fig. 2a). These Hill coefficient values are characteristic of allosteric enzymes and suggest that the enzyme has 2–3 identical sites for the substrate probably because it was composed of 2–3 subunits. The values of kinetic parameters (Vmax, Km, and Hill scope) are displayed on Table 1. When the cells were treated with higher concentrations of H_2_O_2_ (50 µM to 1000 µM) the sigmoid behavior turned into a hyperbolic one (Fig. 2b). Furthermore, H_2_O_2_ treatment affected the Vmax, but not Km of AChE, respect to control (Table 1).

**Table 1:** Kinetic parameters of AChE in SH-SY5Y cells after 24 h treatment with H_2_O_2_.

**+ H**_**2**_**O**_**2**_**(µM)**	**Vmax OD/m/mg**	**Km (µM)**	**Curve type**	**Hill Scope**
**Control**	0.039 ±0.0011	135 ± 20	Sigmoidal	2.95 ± 0.4
1	0.071 ± 0.0028 P<0.001	232 ± 31 (ns)	Sigmoidal	2.50 ± 1.5
50	0.061 ± 0.004 P<0.001	195 ± 25 (ns)	Hyperbola	–
100	0.075 ± 0.0032 P<0.001	218 ± 14 (ns)	Hyperbola	–
400	0.084 ± 0.006 P<0.01	280 ± 11 (ns)	Hyperbola	–
1000	0.062 ± 0.004 P<0.001	172 ± 24 (ns)	Hyperbola	–

* Control condition is referred to untreated cells. Results were obtained from the evaluation with the SigmaPlot and represent data from two different cultures each one performed in triplicate. ns: non-significance.

### 3.3 Effect of H_2_O_2_ on AChE activity in control cell extracts

**Fig. 3:**
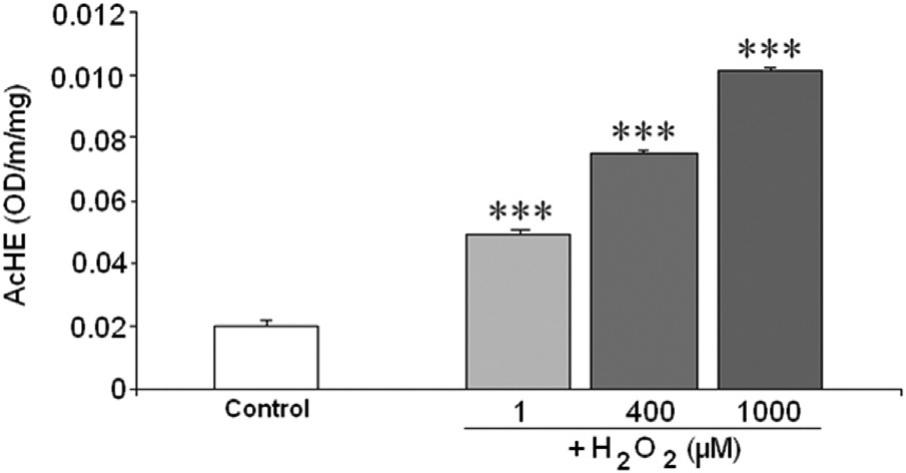
Detection of acetylcholinesterase (AChE) activity of control cell extracts from SH-SY5Y cells as enzyme source treated or not with H_2_O_2_ (1, 400 and 1000 μM) during 30 min·H_2_O_2_ was added to the AChE extract previously obtained from untreated cells. AChE activity was measured by the Ellman's methods using acetylthiocholine (250 μM) as substrate. ***P<0.001.

The effect of H_2_O_2_ on AChE activity was measured in the extracts obtained from control cells in order to know the way in which H_2_O_2_ affects AChE activity on the enzyme without any possible additional effect mediated by the cells. With this experiment we were able to discount the effect of H_2_O_2_ on AChE levels, and the effect of other substances generated inside the cell as a consequence of the toxic action of H_2_O_2_ (e.g. other ROS or Aβ among others). At the beginning and 15 min after the addition of H_2_O_2_ to control cell extracts there was non-significant change in AChE activity (data not shown) respect to control. However, after 30 min of H_2_O_2_ exposure, the enzyme activity increased, confirming the direct effect of H_2_O_2_ on the structure of the enzyme. Furthermore, this experiment indicated that the effect was not immediate, but H_2_O_2_ needed time to induce it. Moreover, the effect displayed by H_2_O_2_ in this case, and in contrast to the previous experiment using treated cells (Fig. 1), was dose-dependent (Fig. 3). This difference detected between both experiments suggested that other mechanism could be involved when the cells are treated with H_2_O_2_ apart from the one demonstrated.

### 3.4 Impact of H_2_O_2_ on AChE levels

**Fig. 4:**
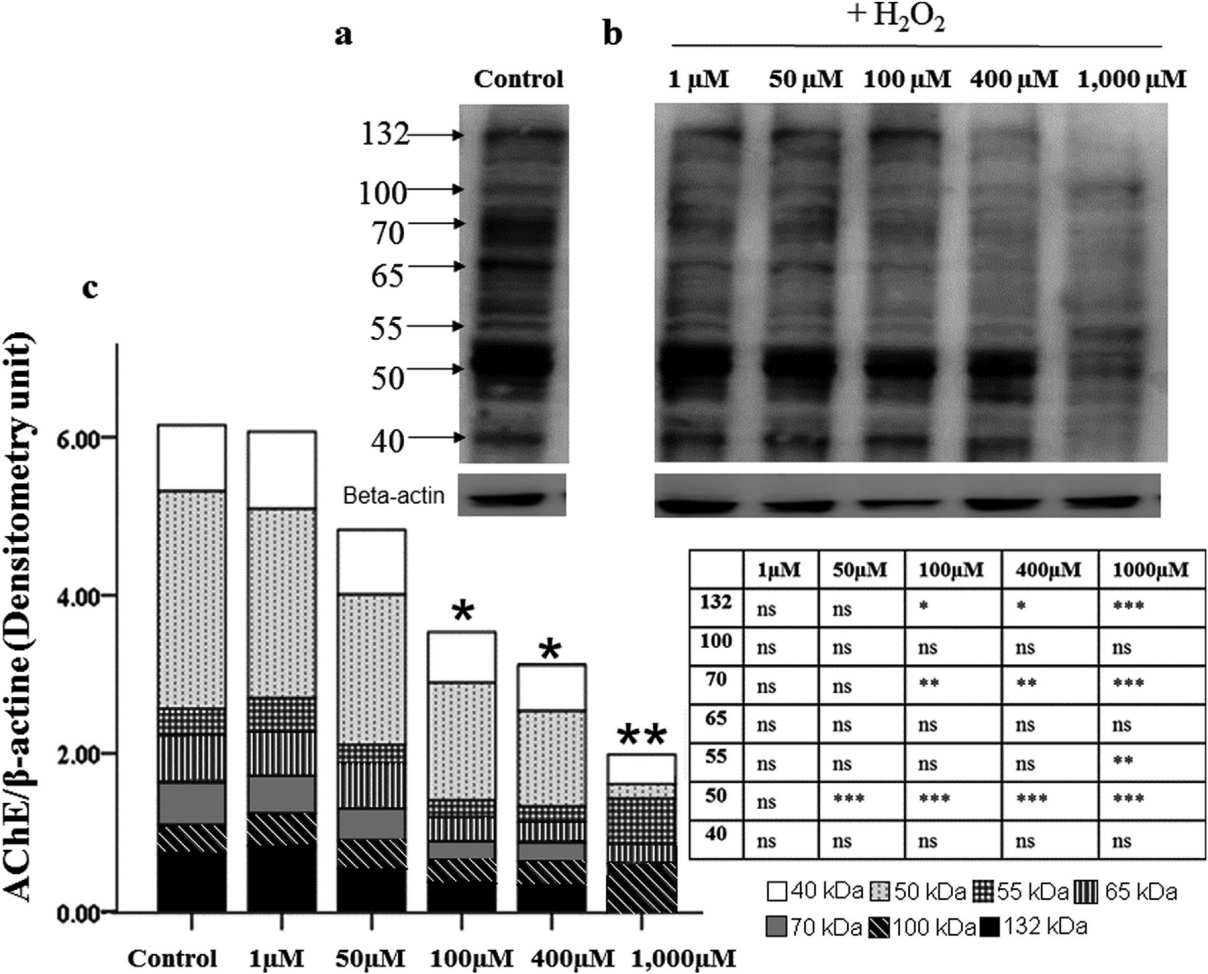
Immunoblots of acetylcholinesterase (AChE) using SH-SY5Y cells extracts as protein source prepared after 24 h treatment with H_2_O_2_. Control condition (C) is referred to untreated cells. **(a)** Immunoblots of control cells with different bands due to the characteristic alternative splicing of AChE gene. **(b)** Immunoblots of cells treated with increasing concentrations of H_2_O_2_ (1, 50, 100, 400 and 1000 µM). **(c)** Quantification of the immunoblots. The results were normalized against beta actin. All isoforms are represented in a stacked column chart from the heaviest (132-kDa) to the smallest (40-kDa). Results of three different cultures each one performed by duplicated. Statistical signification is shown in the table (Bonferroni test). ns= non-significant; *P<0.05; **P<0.01; ***P<0.001.

Western blot analysis allowed us to know the levels and isoforms profile of AChE from control undifferentiated SH-SY5Y cells and their possible changes after H_2_O_2_ treatment. Control samples, without any treatment, presented several bands weights of 132-, 100-, 70-, 65-, 55-, 50- and 40-kDa (Fig. 4a)·H_2_O_2_ induced a clear dose-dependent decrease in the levels of AChE bands (Fig. 4b) judging by the lower bands density in the samples from treated cells.

The densitometry values of each band (expressed as arbitrary densitometry unit respect to β-actin), as well as the total levels obtained by adding the densitometry values of each band from each condition are displayed in Fig. 4c. In this figure it can be observed how the total AChE levels were significantly reduced by H_2_O_2_ from 100 to 1000 µM respect to control sample·H_2_O_2_ at 50 µM significantly decreased the 50-kDa band. The 132- and 70-kDa bands decreased from 100 µM of H_2_O_2_ disappearing at 1000 µM (P<0.001 vs. control). Oppositely, H_2_O_2_ at 1000 µM increased the 55-kDa bands (P<0.01). The statistical significations of individual bands are displayed in the table inserted in Fig. 4c.

### 3.5 Contribution of isoforms to the total AChE content

The contribution of each particular isoform to the total AChE protein would give a clear idea of the isoform profile resulting from the alternative splicing of this enzyme. The percentages of the isoforms are presented in Table 2. The 50-kDa band seemed the more susceptible to H_2_O_2_ treatment since it changed respect to control sample from 50 μM. The other bands were not modified until 400 µM (100-kDa) and 1000 μM of H_2_O_2_ (132-kDa). In fact, such treatment with H_2_O_2_ (1000 μM) clearly modified the balance between isoforms. Bands at 100- and 55-kDa significantly increased (5.3 and 4.95 times, respectively) their contribution to total protein compared with the control cells, while 50-kDa band decreased (4.6 times). Besides, the 132- and 70-kDa bands disappeared.

**Table 2:** Contribution of isoforms to the total AChE content in SH-SY5Y cells after 24 h treatment with H_2_O_2_.

	**+ H**_**2**_**O**_**2**_**(µM)**					
**Isoform**	**Control**	**1**	**50**	**100**	**400**	**1000**
**132**	11.19 ± 0.4	13.20 ± 3.3 ns	13.71 ± 2.6 ns	10.92 ± 2.1 ns	11.00 ± 1.3 ns	0.00 P<0.001
**100**	5.68 ± 0.5	6.50 ± 1.3 ns	6.73 ± 2.3 ns	7.38 ± 1.0 ns	9.32 ± 0.8 P=0.5	30.45 ± 3.4 P<0.001
**70**	8.25 ± 1.0	7.84 ± 1.0 ns	8.06 ± 1.2 ns	6.61 ± 2.1 ns	7.69 ± 0.4 ns	0.00 P<0.001
**65**	8.68 ± 3.1	8.83 ± 1.0 ns	11.13 ± 2.7 ns	8.96 ± 0.3 ns	8.44 ± 1.1 ns	14.5 ± 2.1 ns
**55**	5.15 ± 0.3	6.84 ± 2.7 ns	4.50 ± 0.9 ns	6.51 ± 1.3 ns	6.25 ± 1.4 ns	25.51 ± 3.3 P<0.001
**50**	47.05 ± 1.6	41.54 ± 4.1 ns	39.81 ± 0.5 P=0.03	40.68 ± 1.0 P=0.05	38.73 ± 1.0 P=0.011	10.25 ± 1.9 P<0.001
**40**	9.21 ± 7.7	9.22 ± 7.3 ns	10.09 ± 8.1 ns	12.72 ± 10.6 ns	12.76 ± 10.7 ns	14.07 ± 11.9 ns

† Control condition is referred to untreated cells. The corrected data (densitometry unit respect to beta-actin) of all bands were added up and considered as the 100% of total AChE protein. The specific contribution of each band to global AChE content was expressed as the percentage of each band (band densitometry x 100/ total densitometry AChE protein). ns: non-significance.

### 3.6 PCA results

**Fig. 5:**
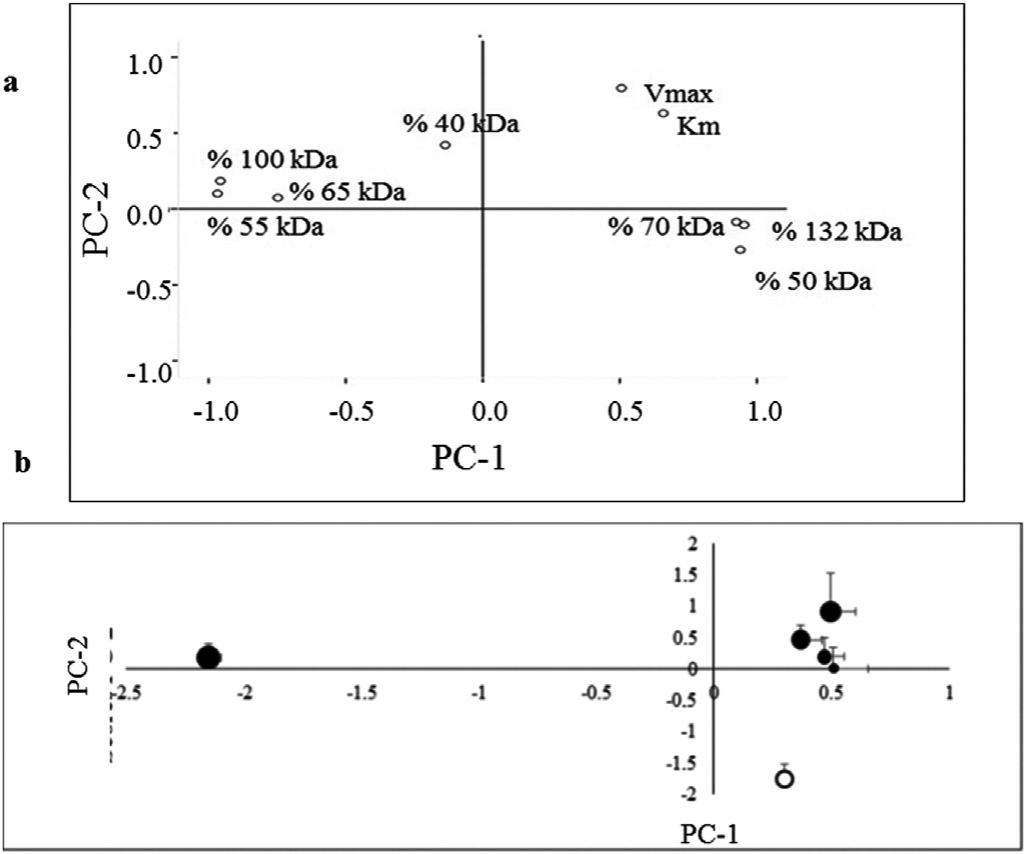
Principal component analysis (PCA) results. **(a)** Loading PCA plot for nine variables: the contribution (%) of the AChE seven isoforms (132-, 100-, 70-, 65-, 55-,50- and 40-kDa), and the kinetic parameters (Vmax and Km). Dot lines at 0 in the PC-1, and PC-2 scales mean no contribution of each variable to the specific PC. This figure shows the different correlations between the different variables considered taking into account the two PCA components. **(b)** Scatter plot using nine variables: the contribution (%) of the AChE seven isoforms (132-, 100-, 70-, 65-, 55-, 50- and 40-kDa), and the kinetic parameters (Vmax and Km). PC-1 and PC-2 accounted for 64.06% and 14.96% of the variance, respectively. Dot lines at 0 in the PC-1 and PC-2 scales means no explanation by the specific PC to the variability found in each treatment.  Control cells;  1 μM H_2_O_2_;  50 μM H_2_O_2_;  100 μM H_2_O_2_;  400 μM H_2_O_2_;  1000 μM H_2_O_2_.

Principal component analysis (PCA) was conducted to ascertain possible relationships between bands, together with the link between AChE activity and isoform profile. The two isoforms present in the brain (AChE-R and AChE-S) could appear as G1 forms, but also associated as G2 or higher structures, and hence some of the bands that we detected in the western blot are likely to be related to one another (Table 3, Fig. 5a and b).

**Table 3:** Rotated component matrix for the analysis of the relation between AChE isoforms (%) and kinetic parameters (Vmax and km).

**Variable**	**PC-1**	**PC-2**
Vmax	0.506	0.796
Km	0.658	0.630
132 (%)	0.925	−0.085
100 (%)	−0.957	0.184
70 (%)	0.953	−0.104
65 (%)	−0.748	0.074
55 (%)	−0.968	0.101
50 (%)	0.939	−0.268
40 (%)	−0.137	0.420

‡ Factors were extracted with the principal component analysis (PCA) using the Varimax rotation. PC, principal component.

Nine different variables were evaluated: the contribution (%) of the seven different isoforms (132-, 100-, 70-, 65-, 55-, 50- and 40-kDa), and two related to the kinetic parameters (Vmax and Km). The PCA created two components which explained the 79.01% of the total variance in the data set (Fig. 5a). First component (PC-1) accounted for 64.06% of the variance. It correlated strongly and positively with 132-, 70- and 50-kDa bands (see lower right quadrant of the plot) and their values indicated in Table 3 were close to 1. On the other hand, there were 100-, 65- and 55- kDa bands, with values close to −1, located on the left side of the plot (Fig. 5a), which correlated negatively with the other bands.

Second component (PC-2) explained 14.96% of the total variance. It correlated positively with both kinetic parameters, Km and Vmax, and also with the 40-kDa band, appearing at the top of the plot with the 40-kDa band alone in the middle of the plot (Fig. 5a). Summarizing, the PCA scatter plot divides the different bands in two groups of three bands each, appearing the 40-kDa band in the middle of both. Moreover, the kinetic parameters seemed to be more related to the 132-, 70- and 50-kDa bands. The PCA scatter plot (Fig. 5b) indicates the position of the studied groups in a graph compiled with two components (PC-1 and PC-2). It showed how the control cells were located on the positive part of PC-1 but the negative part of PC-2, while with growing H_2_O_2_ concentrations (from 1 µM to 400 µM), PC-2 positive values, shifted to more positive positions. Finally, the position of the treatment with 1000 µM of H_2_O_2_ was completely different from the other treatments, situated on the negative values of PC-1.

### 3.7 Effect of H_2_O_2_ on cytochrome c release

**Fig. 6:**
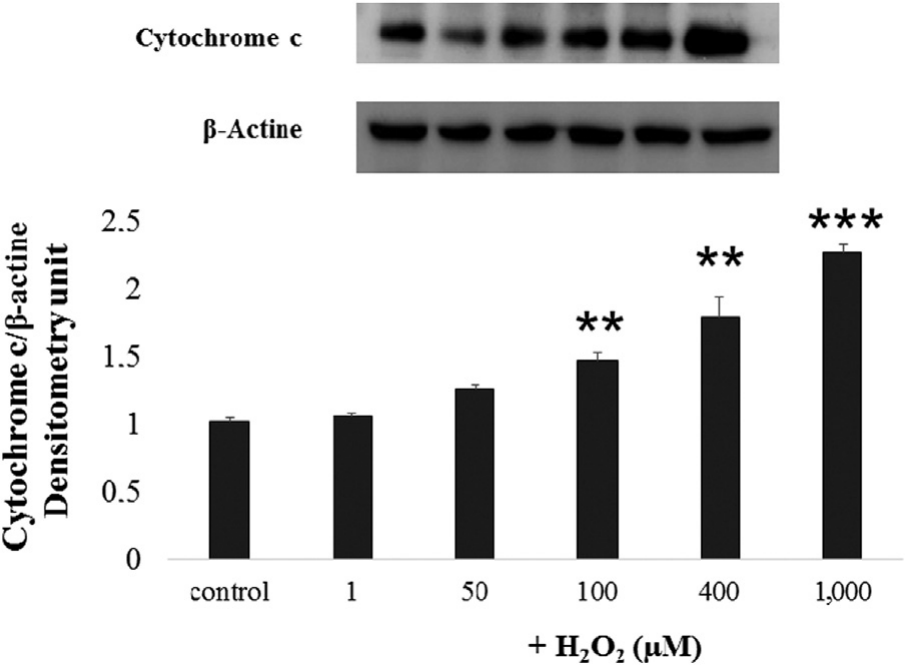
Immunoblots of cytosolic cytochrome c using SH-SY5Y cells extracts as protein source, prepared after 24 h treatment with H_2_O_2_. Control condition (C) is referred to untreated cells. The results were normalized against beta actin. The extracts were obtained from three different cultures, each one performed by duplicated. **P<0.01; ***P<0.001.

In order to establish the relation between the effect of H_2_O_2_, AChE and apoptosis, the levels of cytochrome c in cytosol were tested by immunoblot. The presence of cytochrome c in the cytosol increased in parallel to H_2_O_2_ concentrations tested, being significant for concentrations of H_2_O_2_ ≥100 µM (Fig. 6).

## 4 Discussion

This paper offers novel information about the H_2_O_2_ (ROS) mechanism operating on AChE from SH-SY5Y human neuroblastoma cells. The study has the strength of testing five increasing concentrations of H_2_O_2_ and assessing both the activity and the protein levels of AChE as well as cytochrome c release. Present findings are interesting given the presence of oxidative stress, as well as the implication of AChE in the early stages of neurodegenerative diseases [27], [28].

### 4.1 H_2_O_2_ induced AChE activation

The present results show the activation of AChE when the cells were treated with H_2_O_2_. Such activation, triggered by oxidative stress, would imply an additional damage to the neurons beside the neuronal death. Activator effect of oxidative stress promoted by H_2_O_2_ or other pro-oxidant substances on AChE has been previously found by several authors in different cell lines [17], [18], [29], [30]. Jiang et al. [30] detected an increase in AChE activity after H_2_O_2_ insult in PC12 cells. These authors attributed the increase in AChE activity to oxidative stress because Glutathione, which is involved in cellular redox homeostasis, inhibited the increase of AChE activity. These results agree with ours; however, these authors did not study the mechanism by which oxidative stress induces AChE activation. Molochkina et al. [18] found that H_2_O_2_ at low concentrations increases the membrane AChE activity from erythrocytes; while H_2_O_2_ at high concentrations inhibits AChE activity. Such variation of AChE activity was explained by membrane modifications promoted by H_2_O_2_ as lipid peroxidation. Despite we found in our model lipid peroxidation induced by H_2_O_2_ [31], we discarded this mechanism considering data from the experiment with control cell extracts. In this control cells there is not lipid peroxidation, but H_2_O_2_ treatment increased AChE activity. Hence, lipid peroxidation could potentiate the effect of H_2_O_2_ on AChE activity but it is not the main cause in our case.

The increase in AChE activity observed in SH-SY5Y cells with H_2_O_2_ treatments seems to be mediated by a direct effect on the structure of the enzyme and not on the enzyme synthesis. This mechanism was assessed in the light of the following findings in this study: a) the kinetic parameters studied displayed a change from sigmoidal to hyperbolic behavior, impacting on the Vmax and not on the Km; b) the direct effect of H_2_O_2_ on AChE of control cell extracts showed a sharp increase of activity, which was dose- and time- dependent; and c) AChE levels were much lower in the presence of higher H_2_O_2_ concentrations, rather than increasing, as was expected in view of the increased activity of this enzyme. These results disagree with those of Zhang et al. [17] which found that during the apoptosis induced by H_2_O_2_ in 293 T cells there was increase in AChE expression via transcriptional activation of c-Jun N-terminal kinase (JNK). This contradictory effect could be due to a different response to H_2_O_2_ in the different cell lines 293 T (kidney human cells) and SH-SY5Y (neuroblastoma human cells).

The activator effect of H_2_O_2_ on control cell extracts was found to be dose-dependent, but no such effect was observed in cells treated for 24 h, where AChE activity was high at H_2_O_2_ concentrations of 1 µM and kept high until 1000 μM without a dose-dependent increase. This could be because the total AChE content decreased due to H_2_O_2_ treatment, and the enzyme activity was expressed depending on total protein content; hence, the ratio total proteins respect to AChE protein was modified by the treatment. However, in the experiment with control cell extract AChE protein level is the same in all H_2_O_2_ treatments, which means that total proteins/AChE protein ratio was unchanged.

In the case of the kinetic parameters, the sigmoidal behavior of control cells suggests that AChE of SH-SY5Y cell line is an allosteric enzyme. Furthermore, the positive value of the Hill coefficient is indicative that the substrate (ACh) presents positive cooperativity. Moreover, the value of the Hill coefficient can be related to the number of active sites of the enzyme, which in this case would agree with the subunit number found. Our control cells showed a Hill coefficient between 2 and 3, which could result from the mixture of monomers, dimmers and tetramers characteristic of this protein. On the other hand, the change observed in enzymatic behavior (Fig. 2c) suggests that H_2_O_2_ acts as an allosteric activator of AChE in SH-SY5Y cells. Furthermore, H_2_O_2_ would seem to act as a V-type heterotrophic effector, since it modified Vmax but not the Km. This behavior could be mediated by oxidation of some amino acid from the active site. In this sense, Schallreuter et al. [16] observed that at concentrations lower than 1000 µM, H_2_O_2_ caused the activation of AChE via oxidation of methionine, cysteine and tryptophan. Other possibility could be the different AChE isoform composition induced by H_2_O_2_ in SH-SY5Y cells. In this sense, Bond et al. [29] treating astroglia cells with tert-butyl hydroperoxide, an oxidative stress generator, found that the increase of AChE activity promoted by this agent was via switching of mRNA between AChE-T and AChE-R isoforms.

### 4.2 H_2_O_2_ changed the AChE levels

Very often the studies conducted to ascertain the alterations of AChE are based on enzymatic activity determinations. Conversely, some authors highlight the importance of measuring AChE levels too, since the two parameters (activity and levels) do not always vary in the same direction [1].

The AChE bands detected in SH-SY5Y cells agree with those found by Darreh-Shori et al. [11] in the cortex and cerebrospinal fluid of AD patients, which could be explained because SH-SY5Y cells are nervous cells as has been mentioned above. In the present paper it has been quantified those bands which displayed changes after H_2_O_2_ treatment. The role of this isoform diversity is not well understood. The tetramer form (more than 200-kDa) was not detected in the present work perhaps because, as noted, protein content was measured under reduced conditions. The 132- and 100-kDa bands were considered dimer-trimer, and the smaller forms 70-, 65-, 55- and 50-kDa, monomers. Finally, the 40-kDa band could be defined as a proteolysis product of the enzyme as suggested by Berson et al. [8].

The PCA test showed a strong correlation between 132-, 70- and 50-kDa bands, quite close to the kinetic parameters (Vmax and Km) (Fig. 5a), suggesting that these three isoforms are more related to the catalytic activity. Moreover, Darreh-Shori et al. [11] report that 50-kDa isoform is included in heavy complexes because it dramatically increases when the experiment is conducted under reduced conditions. On the other hand, PCA test kept apart the 100-, 65- and 55-kDa isoforms, probably because they are also related to other functions (e.g. apoptosis and synaptogenesis, among others). Nonetheless, it is likely that there is also catalytic activity in the other three isoforms, since in our experiments 1000 μM of H_2_O_2_ kept the activity high while the bands of 132- and 70-kDa disappeared. H_2_O_2_ treatment modified AChE isoform profile from SH-SY5Y cell line. Although the consequence of this change is not well-known, AChE composition inside the cells could be important as depends on the physiological needs [32].

The treatment with increasing H_2_O_2_ concentrations gradually reduced the total AChE contents. The fall in AChE levels due to the treatment could mean that H_2_O_2_: a) caused degradation of the enzyme; b) induced AChE release; c) reduced the available amount of Ach and/or cellular death by apoptosis.

Degradation of the enzyme as an isolated event did not seem the most probable mechanism, since the 40-kDa band was not significantly affected as compared to the control (Fig. 4c). AChE release has been reported by Hicks et al. [10], although the mechanism for this release is not well understood. Alternatively, AChE decrease due to cellular death is other possibility. In fact, our group [31] observed that a 24 h treatment with 400 µM of H_2_O_2_ in SH-SY5Y promote cell death by apoptosis and necrosis. Furthermore, we have found in the present study that H_2_O_2_ promoted significant increase of cytochrome c in the cytosol from 100 μM to 1000 μM.

Regarding the last possibility, the amount of Ach could decrease due to increased degradation, lower synthesis, or cell death.

### 4.3 H_2_O_2_ modified AChE isoform profile inducing apoptosis

The involvement of AChE in apoptosis has been fairly well established [33]. Actually, we found a positive correlations between the cytochrome c and the AChE 100- and 55-kDa isoforms (percentage value) (*P*<0.0001; *P*=0.002; respectively). The implication of 55-kDa isoform of AChE in apoptosis has been suggested by Xie et al. [34]. The possibility that H_2_O_2_ selectively affects cholinergic neurons, inducing their death by apoptosis and promoting cholinergic system dysfunction must be evaluated in future experiments.

In the present experiments, considerable changes were observed in all parameters measured following an insult of H_2_O_2_ 1000 μM. Future studies would do well to assess the chronic effect of oxidative stress on AChE in animal models of neurodegenerative diseases.

## 5 Conclusions

In conclusion, present results suggest that: 1) oxidative stress promotes major disturbances in the levels of AChE, increasing its activity but oppositely reducing its levels, which could have important consequence in cholinergic transmission in SH-SY5Y cells (**Fig. 7; graphical abstract**); 2) H_2_O_2_ promotes cellular death by apoptosis, which together with AChE alterations would imply an additional mechanism in the hazardous effect of oxidative stress in this cell line. All these results demonstrate for the first time the possible mechanism by which an oxidant agent activate AChE in human neuroblastoma cells, and makes aware of the importance of studying the impact of antioxidant agents treatment as a suitable strategy to decrease ROS levels and consequently to block AChE activity and isoform profile alterations.

## Conflict of interest

The authors declare that they are not conflicts of interest.
